# High prevalence of carbapenem-resistant *Pseudomonas aeruginosa* and identification of a novel VIM-type metallo-β-lactamase, VIM-92, in clinical isolates from northern China

**DOI:** 10.3389/fmicb.2025.1543509

**Published:** 2025-02-26

**Authors:** Linbo Zhao, Jiekun Pu, Yunning Liu, Heng Cai, Meijuan Han, Yunsong Yu, Jianhua Tang

**Affiliations:** ^1^Hebei Key Laboratory of Neuropharmacology, Hebei North University, Zhangjiakou, China; ^2^Department of Pharmacy, The First Affiliated Hospital of Hebei North University, Zhangjiakou, China; ^3^Department of Infectious Diseases, Sir Run Run Shaw Hospital, Zhejiang University School of Medicine, Hangzhou, China; ^4^Department of Pharmacy, Cangzhou Central Hospital, Cangzhou, Hebei, China; ^5^Institute of Disaster and Emergency Medicine, Tianjin University, Tianjin, China

**Keywords:** *Pseudomonas aeruginosa*, epidemiological investigation, metallo-β-lactamase, VIM-92, plasmid

## Abstract

Carbapenem-resistant *Pseudomonas aeruginosa* (CRPA) has become a serious global health concern due to the limited treatment options. The primary resistance mechanism in CRPA involves the production of metallo-β-lactamases (MBLs), making MBL-producing *P. aeruginosa* a significant component of CRPA cases. To understand the prevalence of CRPA in hospitals in northern China, we conducted a preliminary screening and identification of CRPA in 143 clinical isolates of *P. aeruginosa* collected from various departments of a tertiary hospital between 2021 and 2023, analyzing CRPA resistance trends in certain regions of northern China during this period. We identified 71 CRPA isolates that exhibited high carbapenem resistance and phylogenetic tree analysis revealed that ST244 CRPA isolates had widely spread across various departments of the same hospital over three consecutive years. We also identified two VIM-producing isolates, PJK40 and PJK43, both of which carried the same novel VIM-type metallo-β-lactamase, VIM-92, encoded by a newly identified gene, *bla*_VIM-92_, closely related to *bla*_VIM-24_. *bla*_VIM-92_ was embedded in class 1 integrons within the Tn*1403* transposon. The *bla*_VIM-92_-carrying plasmid, pPJK40, was found to resemble the pJB37 megaplasmid. The expression of VIM-92 and VIM-24 in DH5α and PAO1 revealed similar effects of the MICs of β-lactams, except for aztreonam. The high prevalence of CRPA in clinical settings, and the identification of VIM-92, highlights the urgent need for ongoing surveillance of CRPA and emerging MBL variants in *P. aeruginosa*.

## 1 Introduction

*Pseudomonas aeruginosa* (*P. aeruginosa*) is a Gram-negative, opportunistic pathogen commonly associated with hospital-acquired infections, especially in immunocompromised patients and individuals with cystic fibrosis (Qin et al., 2022). Carbapenem antibiotics are often the first-line treatment for *P. aeruginosa* infections due to their broad antibacterial spectrum, potent activity, and rapid onset (Takahashi et al., 2021). However, the global CRPA increase has become a significant health concern. *P. aeruginosa* can acquire resistance to carbapenem through various mechanisms, producing carbapenemase enzymes encoded by carbapenemase genes as a primary resistance pathway (Tenover et al., 2022). To date, class A, B, and D carbapenemases have been identified in *P. aeruginosa*, with class B metallo-β-lactamase (MBL) enzymes, such as Verona Integron-encoded Metallo-β-lactamase (VIM), imipenemases (IMP), and New Delhi metallo-β-lactamase (NDM), being the most prevalent (Park and Koo, 2022). MBLs can hydrolyze most β-lactams, except monobactams, but remain unaffected by novel β-lactamase inhibitors such as avibactam, vaborbactam, relebactam, and nacubactam (Jean et al., 2019). Consequently, therapeutic options for infections caused by MBL-producing *P. aeruginosa* are substantially limited. In addition, bacterial secretion systems played a significant role in the infection and pathogenesis of CRPA. The virulence of CRPA was closely associated with its encoded secretion systems, including type I to type VI secretion systems (Wood et al., 2015). Among these, the type III secretion system (T3SS) was the most complex and virulent secretion system in CRPA, primarily involving four virulence factors: *exoY*, *exoT*, *exoU*, and *exoS*.

The VIM-type enzyme is the most prevalent MBL in Europe, with over 80 variants identified to date (Botelho et al., 2019). According to relevant literature, the detection rate of CRPA in northern China is significantly higher than in other regions, with VIM-type enzymes being more frequently detected than other types of MBLs (Wu et al., 2024). Among these, *bla*_VIM-2_ is the most frequently reported carbapenemase-encoding gene in *P. aeruginosa* spp. In recent years, isolates carrying novel VIM-type alleles have continuously been identified. According to recent literature, *bla*_VIM-84_, a novel VIM-type MBLs, was first reported in the IncP-2 megaplasmid of *P. aeruginosa* (Wang et al., 2023). This indicates the potential for the transfer and spread of *bla*_VIM-84_ in *Pseudomonas* species. In another study, researchers characterized a *Pseudomonas monteilii* (*P. monteilii*) isolate carrying *bla*_VIM-84_ for the first time, which conferring resistance to β-lactams (Tu et al., 2024). Genome analysis revealed that *bla*_VIM-84_ was located within a class I integron, with Tn*3* surrounding. This structure suggested potential for dissemination. MBL-encoding genes are typically integrated within class I integrons and are transmitted via mobile genetic elements, which commonly embed *bla*_VIM_ genes into genetic cassettes, facilitating their widespread dissemination (Zhang et al., 2023).

Zhangjiakou, in China, the location of this study, occupies a unique geographic position at the intersection of Beijing, Tianjin, Hebei, and Inner Mongolia, which can contribute to different sources of infection and epidemiological patterns. The First Affiliated Hospital of Hebei North University, a tertiary hospital in Zhangjiakou, serves as a central healthcare facility, providing critical data to understand antimicrobial resistance trends in northern China. These data support regional and interregional public health collaborations and comparative studies on *P. aeruginosa* resistance. For this study, we collected 143 *P. aeruginosa* isolates from clinical specimens submitted by various departments at our hospital between January 1, 2021, and December 31, 2023. Following culture, identification, and antimicrobial susceptibility testing, we analyzed the transmission dynamics within the hospital and resistance patterns of CRPA. After excluding duplicate isolates from the same patient and sample size, we identified 71 CRPA isolates. Among these, two were VIM-producing *P. aeruginosa*, PJK40 and PJK43. Whole-genome sequencing revealed that the genetic sequences of these two isolates were identical, and both of them carried a new variant of VIM-2, VIM-92, with the difference of isolation sources. Therefore, to avoid redundancy, we selected PJK40, strained from lavage fluid, further analyzed the genetic characterization of this isolate, and evaluated the effect of VIM-92 on antibiotic resistance.

## 2 Materials and methods

### 2.1 Collection and identification of bacteria isolates

Clinical isolates of *P. aeruginosa* were obtained from patient samples at the First Affiliated Hospital of Hebei North University using selective *Pseudomonas* Isolation Agar plates. And then identification was confirmed using matrix-assisted laser desorption ionization-time of flight mass spectrometry (Bruker Daltonik GmbH, Bremen, Germany). Data on 143 *P. aeruginosa* isolates, including the year of detection, department, and source of the isolates, were retrieved from the hospital’s electronic medical records.

### 2.2 Antimicrobial susceptibility determination

The minimum inhibitory concentrations (MICs) of antibiotics were determined using the broth microdilution method. The antibiotics tested included Meropenem (Hanhui Pharmaceutical Co., Ltd., China), Imipenem (Merck Sharp & Dohme Corp., USA), Cefepime (Jiangsu Hengrui Pharmaceutical Co., Ltd., China), Piperacillin (Suzhou Erye Pharmaceutical Co., Ltd., China), Ceftazidime (Guangdong Jincheng Jinsu Pharmaceutical Co., Ltd., China), Tazobactam (Meilunbio, China), Avibactam (MedChemExpress, USA), Aztreonam (Sigma-Aldrich, USA), Ciprofloxacin (Fluka Analytical, USA), Amikacin (Meilunbio, China), and Colistin (Sigma-Aldrich, USA). *P. aeruginosa* isolate ATCC 27853 and *Klebsiella pneumoniae* ATCC 700603 were used as quality control isolates. The diluted cultures were incubated overnight at 37°C, and results were interpreted according to the CLSI performance standards (30th Edition) (Clinical and Laboratory Standards Institute, 2020). According to the CLSI standards, CRPA were defined as *P. aeruginosa* exhibiting resistance to any carbapenem, including imipenem and meropenem. In this study, isolates with a MIC ≥128 were classified as highly resistant isolates (Park and Koo, 2022). The VIM alleles were identified by PCR amplification of the full ORF, using primer pairs VIM-F (5′-tgccgtagaagaacagcaag-3′) and VIM-R (5′-gcaacttcatgttatgccgc-3′). The PCR products were sequenced using Sanger sequencing.

### 2.3 Whole-genome sequencing and bioinformatics analysis

All 71 CRPA isolates were subjected to second-generation sequencing on the Illumina HiSeq platform. Genomic DNA was extracted using the QIAamp DNA Mini Kit (Qiagen, Hilden, Germany) following the manufacturer’s instructions. Sequencing was performed on the Illumina HiSeq platform, and raw reads were assembled using Shovill 0.9.0.^1^ Additionally, two VIM-producing isolates were selected for third-generation sequencing. For these isolates, hybrid assembly that combined Illumina and Nanopore reads was conducted with Unicycler v.0.4.8 (Wick et al., 2017). Multilocus sequence typing (MLST) was determined through PubMLST,^2^ and gene annotation was performed with Prokka v.1.14.6 (Seemann, 2014). ABRicate v.1.0.0^3^ was used for the identification of resistance genes and virulence factors, while sequence alignment against the plasmid was performed using BWA-MEM (Li, 2014). Genetic sequence comparisons and visualizations were generated using Easyfig 2.2.5 and BRIG-0.95 (Alikhan et al., 2011; Sullivan et al., 2011). Based on the annotation results from Prokka, the genomic data of 71 CRPA isolates were selected for pan-genome analysis using Roary v.3.13.0 (Page et al., 2015). Phylogenetic tree construction was conducted with FastTree v.2.1.11 (Price et al., 2009) using default parameters, and the tree was visualized and annotated with features using ChiPlot (Xie et al., 2023).

### 2.4 Conjugation of plasmids

Conjugation experiments were conducted using clinical isolates as donors and a rifampin-resistant derivative of *P. aeruginosa* PAO1 as the recipient. The selection was performed with rifampicin (300 μg/mL) and ceftazidime-avibactam (16 μg/mL). The donor and recipient bacterial colonies were cultured in 2 mL of Luria-Bertani (LB) medium and shaken at 37°C for 4 h. The isolates were mixed in LB medium in a 1:1 ratio (100 μL each). A 20 μL aliquot of the mixture was placed on a sterile 0.22-μm pore-size Millipore filter of 0.22-m size on a Mueller-Hinton (MH) agar plate and cocultured at 37°C overnight. The bacterial lawn formed on the filter was harvested, resuspended in 200 μL of LB broth, and seeded on agar containing selective antibiotics. After overnight incubation at 37°C, colonies that grew in the selective plates were confirmed by PCR amplification.

### 2.5 Cloning procedures

Plasmid pGK1900 was designed to express MBL genes. In brief, the oriT and traJ region from plasmid pCasPA (Chen et al., 2018), and the GmR region from plasmid pEX18Gm (Hoang et al., 1998), were amplified and recombined into the broad-host-range plasmid pACRISPR (Chen et al., 2018), which carries the pRO1600 oriV and T7 promoter. The final plasmid, pGK1900, was constructed.

The *bla*_VIM-92_ gene, along with its upstream predicted promoter (identified using Softberry),^4^ was amplified from the clinical isolate PJK40 and cloned into pGK1900 using the Hieff Clone Plus One Step Cloning Kit (Li et al., 2022). The resulting VIM-expressing plasmids were introduced into *Escherichia coli* DH5α via chemical transformation and into *P. aeruginosa* PAO1 by electroporation.

### 2.6 Data analysis

SPSS 20.0 and WHONET v5.6 software (WHO Collaborating Centre for Surveillance of Antimicrobial Resistance, Boston, MA, USA) were used to analyze the data. The counting data were expressed as the number of cases (n) and rate (%).

## 3 Results

### 3.1 Collection and distribution of CRPA

Between 2021 and 2023, 143 *P. aeruginosa* isolates were detected, of which 71 were identified as CRPA, excluding duplicate isolates from the same patient, resulting in a CRPA detection rate of 49.65%. Statistical analysis indicated a significant downward trend in the CRPA detection rate over the 3 years (*Z* = 1.850, *p* = 0.0174) (Supplementary Table S1). These 71 CRPA isolates were distributed between various departments, with the highest proportion of other departments (20 isolates, 28.17%), followed by the Respiratory Department (18 isolates, 25.35%) and the International Medical Department (14 isolates, 19.72%) (Supplementary Table S2). And the primary source of CRPA specimens was sputum, accounting for 55 isolates (77.46%), followed by lavage fluid and other sources, each with five isolates (7.04%) (Supplementary Table S3).

### 3.2 MIC for CRPA

The CRPA isolates exhibited high resistance rates to carbapenems, with imipenem (98.59%) and meropenem (78.87%). Resistance to most cephalosporins (ceftazidime, cefepime), β-lactams (aztreonam), enzyme inhibitor complexes (piperacillin/tazobactam, ceftazidime/avibactam) and quinolones (ciprofloxacin, levofloxacin) exceeded 49%, indicating a multidrug-resistant profile. In contrast, the isolates remained relatively susceptible to amikacin and polymyxins, with resistance rates below 34%, suggesting that these agents may still be effective treatment options. During the 3-year period, the resistance rate to piperacillin-tazobactam initially declined but then increased (*p* < 0.05), while resistance to ceftazidime-avibactam, aztreonam, imipenem, ciprofloxacin, levofloxacin, and amikacin showed a decreasing trend (*p* < 0.05). No statistically significant differences were observed in resistance rates to ceftazidime, cefepime, meropenem, and colistin (Table 1). MICs for 71 CRPA isolates are provided in Supplementary Table S4.

**Table 1:** Changes in drug resistance rates of CRPA isolates to commonly used antibiotics from 2021 to 2023.

Department	2021 (*n* = 24)		2022 (*n* = 25)		2023 (*n* = 22)		Total (*n* = 71)		*X* ^2^	*p*
	Isolate count	Proportion (%)	Isolate count	Proportion (%)	Isolate count	Proportion (%)	Isolate count	Proportion (%)		
PTZ	14	58.33	9	36	16	72.73	39	54.93	6.546	0.038
CAZ	16	66.67	11	44	15	68.18	42	59.15	3.679	0.159
CZA	16	66.67	7	28	13	59.09	36	50.7	8.221	0.016
FEP	17	70.83	10	40	16	72.73	43	60.56	2.259	0.323
AZT	17	70.83	14	56	15	68.18	46	64.79	8.442	0.015
IMI	24	100	25	100	21	95.45	70	98.59	6.849	0.033
MEM	22	91.67	15	60	19	86.36	56	78.87	1.342	0.511
CIP	18	75	5	20	12	54.55	35	49.3	15.171	<0.001
LEV	20	83.33	7	28	13	59.09	40	56.34	15.340	<0.001
AK	15	62.5	2	8	7	31.82	24	33.8	16.310	<0.001
COL	1	4.17	2	8	1	4.55	4	5.63	0.409	0.815

PTZ, piperacillin-tazobactam; CAZ, ceftazidime; CZA, ceftazidime-avibactam; FEP, cefepime; AZT, aztreonam; IMI, imipenem; MEM, meropenem; CIP, ciprofloxacin; LEV, levofloxacin; AK, amikacin; COL, colistin.

### 3.3 Genomic features of the 71 CRPA isolates

**Figure 1:**
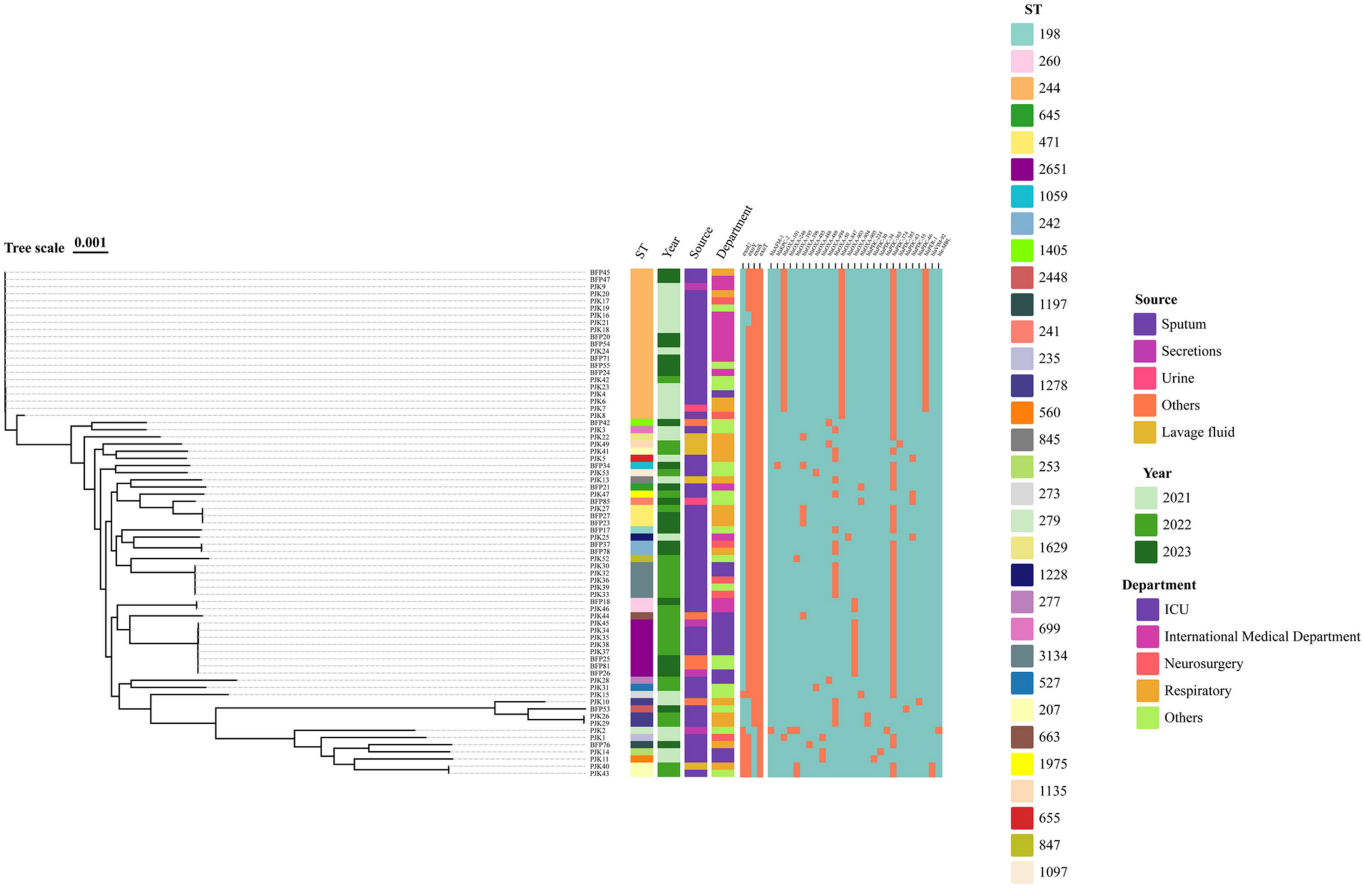
The phylogenetic tree based on the concatenated set of core genes displays the evolutionary relationships among the 71 CRPA isolates. The primary features of CRPA isolates are indicated in various colors, and the presence of T3SS virulence factors and resistance genes is represented by orange.

To further understand the relationships between the 71 CRPA isolates, we constructed a phylogenetic tree based on the nucleotide composition of core genes (Figure 1). MLST is a an unambiguous, portable and nucleotide-based technique for typing bacteria using the sequences of internal fragments of (usually) seven house-keeping genes (Maiden et al., 1998; Spratt, 1999; Urwin and Maiden, 2003). Among the 71 CRPA isolates, 32 sequence types (STs) were identified, with the predominant types being ST244, ST2651, and ST3134, accounting for 29.6% (21/71), 11.3% (8/71), and 7.0% (5/71), respectively. The analysis of STs among the 71 CRPA isolates over 3 years (2021–2023) revealed distinct trends. In 2021, ST244 was the predominant type, accounting for the majority of isolates (24 isolates). In 2022, both ST3134 and ST2651 were the most prevalent types, each represented by 5 isolates. By 2023, ST244 re-emerged as the dominant type, once again constituting the largest proportion of isolates. Notably, ST1278 was prevalent in the first 2 years but disappeared in 2023. Meanwhile, ST60, ST471, and ST2651 began to emerge as significant types starting from 2022. Combined analysis of the ST types, departmental distribution, and isolation years of the 71 CRPA isolates revealed that ST244 CRPA was consistently detected across different departments of the same hospital over a three-year period. In addition, T3SS virulence factor analysis showed that the virulence factor e*xoT* had the highest carriage rate at 100% (71/71), followed by e*xoS* and e*xoY*, both at 90.1% (64/71), while e*xoU* had the lowest carriage rate at 11.3% (8/71). Notably, isolate PJK15 carried all four T3SS virulence factors, which may contribute to its high virulence potential (Supplementary Figure S2).

We predicted resistance genes in the 71 CRPA isolates and identified a total of 54 resistance genes. Among these, four carbapenemase-producing isolates, PJK2, BFP34, PJK40, and PJK43, were detected, carrying the resistance genes *bla*_AFM_, *bla*_KPC_, and *bla*_VIM_. Isolate PJK2 carried the highest number of resistance genes, with a total of 23. The distribution of shared and unique resistance genes is shown in the Supplementary Figure S1.

### 3.4 Identification of VIM-carrying isolates

Bioinformatic analysis of the 71 CRPA isolates revealed mutations in the VIM alleles in two isolates. These two VIM-producing *P. aeruginosa* isolates were identified as PJK40 and PJK43. Sequence analysis revealed that the VIM gene sequences of the two isolates were identical, with the difference of isolation sources. Therefore, PJK40 was selected for further analysis to avoid redundancy. PJK40, carried a novel VIM allele and was isolated from a patient’s bronchoalveolar lavage fluid. Genome sequencing classified PJK40 as sequence type (ST) 207. PCR and sequencing revealed this isolate harbors a novel allele, *bla*_VIM-92_ (GenBank accession: PQ563311). In the amino acid sequence, VIM-24 has an arginine-to-leucine substitution at residue 205 (GenBank accession: HM855205.1), while VIM-92 shows a valine-to-isoleucine substitution at residue 236 (Supplementary Figure S3).

### 3.5 Effects of VIM-24 and VIM-92 on the MICs of β-lactams

To better understand the role *bla*_VIM-92_ in conferring β-lactam resistance, *bla*_VIM-92_ was cloned in the plasmid pGK1900 and transformed into *E. coli* DH5α and *P. aeruginosa* PAO1. The effects VIM-92 on β-lactam resistance to β-lactam differed between DH5α and PAO1 (Table 2).

**Table 2:** MICs of β-lactam antibiotics for the *bla*_VIM-24_ and *bla*_VIM-92_ transformants.

Isolates	MICs of antibiotics (mg/L)							
	MEM	IMI	PIP	AZT	FEP	CAZ	PTZ	CZA
DH5α (pGK1900)	<0.06	0.5	4	0.25	<0.06	1	2	1
DH5α (pGK1900-VIM2 4)	128	16	>128	0.125	1	16	>128	64
DH5α (pGK1900-VIM92)	8	4	128	<0.06	0.5	32	128	16
PAO1 (pGK1900)	2	2	4	4	4	2	8	2
PAO1 (pGK1900-VIM24)	>128	128	>128	8	>128	>128	>128	>128
PAO1 (pGK1900-VIM92)	128	32	128	8	64	>128	128	128

MEM, meropenem; IMI: imipenem; PIP, piperacillin; AZT, aztreonam; FEP, cefepime; CAZ, ceftazidime; PTZ, piperacillin-tazobactam; CZA, ceftazidime-avibactam.

In DH5α, VIM-92 increased the MICs of meropenem, imipenem, piperacillin, cefepime, ceftazidime, piperacillin-tazobactam, and ceftazidime-avibactam by more than 8 times. In PAO1, the MICs of all β-lactams, except for aztreonam, increased by more than 16-fold due to the presence of VIM-92. This is expected, as VIM-92 is an MBL that does not hydrolyze aztreonam. We subsequently cloned *bla*_VIM-24_ using the same method. Antimicrobial susceptibility testing revealed minimal differences in antibiotic resistance effects between VIM-24 and VIM-92.

### 3.6 The characteristics of VIM-carrying plasmids

**Figure 2:**
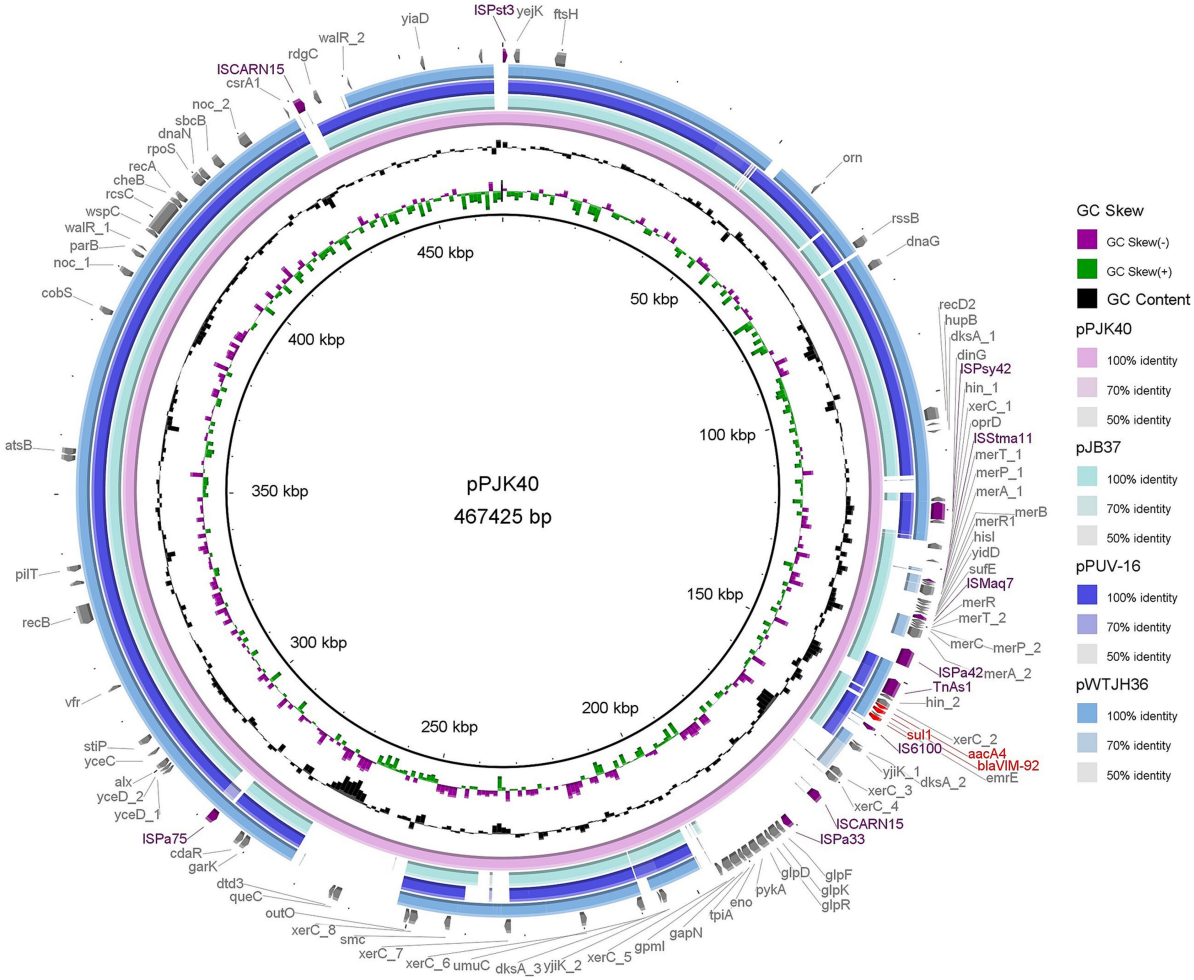
Comparison of pPJK40 with other plasmids. The rings represent the alignment of sequence reads from pJB37, pPUV-16, and pWTJH36 against pPJK40. Both pJB37 and pPUV-16 contain *bla*_VIM-2_, while pWTJH36 contains *bla*_VIM-85_.

Whole-genome sequencing of PJK40 revealed multiple acquired resistance genes, with *bla*_VIM-92_ embedded in the plasmid pPJK40. The pPJK40 plasmid showed query coverage of 82, 81, and 79% with pJB37 (GenBank accession: KY494864.1), pWTJH36 (GenBank accession: CP104591.1), and pPUV-16 (GenBank accession: MT732194.1), respectively (Figure 2). The backbones of these homologous plasmids were similar to those of the IncP-2 plasmid pOZ176, suggesting that they belong to the same megaplasmid family (Zhang et al., 2021). The complete sequence of pPJK40 is 467,425 bp, contains 583 ORFs, and exhibits a GC content of 57%. This plasmid contains a few resistance genes organized into a gene cluster, approximately 144 to 159 kbp, which includes the *bla*_VIM-92_ gene. The differences between pPJK40, pJB37, pWTJH36, and pPUV-16 are mainly due to the presence of insertion sequences (ISs) or integrase-encoding genes, indicating that these plasmids have evolved through multiple insertions and recombination events.

**Figure 3:**
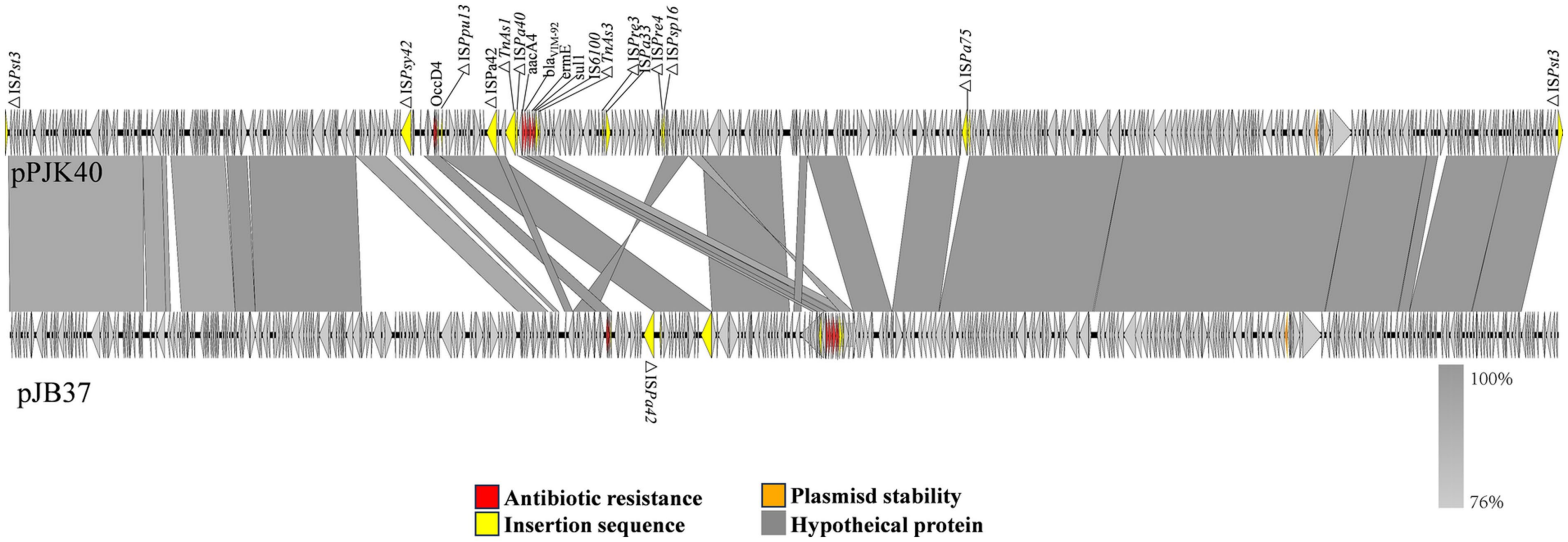
Comparison of pPJK40 and pJB37. The arrow direction indicates the transcription direction of each ORF. The dark yellow arrows represent mobile elements, the red arrows represent resistance genes, and the gray and blue arrows represent predicted ORFs.

Among the homologous plasmids identified in the NCBI database, pPJK40 shows the highest similarity to pJB37, the first bla_VIM-2_-carrying megaplasmid described in *P. aeruginosa* (Botelho et al., 2017). pPJK40 shares 81% query coverage and 99.97% sequence identity with pJB37, according to the BLAST analysis (Figure 3). Several conserved regions were observed between the two plasmids, reflecting a high degree of sequence similarity. The resistance gene cluster in pPJK40 spans 11,365 bp and contains multiple genetic elements and resistance genes, including *bla*_VIM-92_. The strong sequence similarity between this region and the corresponding cluster on pJB37 suggests that these plasmids share similar structural features that support the preservation and transmission of resistance genes. In particular, the insertion sequence downstream of *bla*_VIM-92_ may enhance its mobility, facilitating the spread of resistance genes across bacterial species (Figure 3).

Conjugation assays were conducted to assess the transferability of pPJK40. All three experiments were unsuccessful, suggesting that this plasmid is non-conjugative.

### 3.7 Genetic contexts of *bla*_VIM_ alleles in *Pseudomonas aeruginosa*

**Figure 4:**
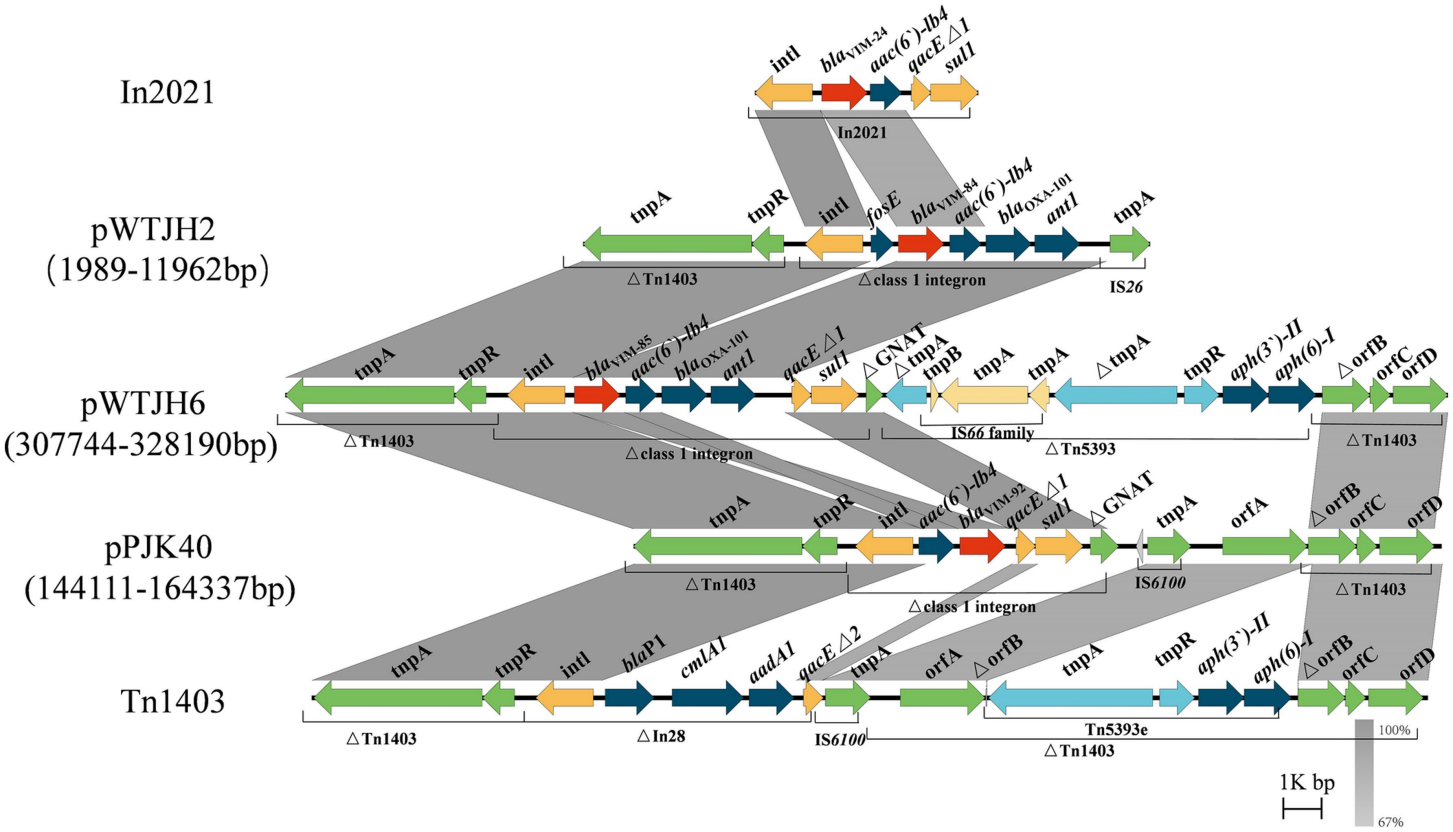
Comparison of the genetic environments of *bla*_VIM_. Shaded regions indicate nucleotide identity (67–100%). Red arrows represent *bla*_VIM_ and other antibiotic resistance genes, shown in dark blue. The yellow, green, and light blue arrows represent structures of mobile elements.

Numerous VIM alleles have been reported in *P. aeruginosa* (Pournaras et al., 2003; Siarkou et al., 2009). In a previous report, sequencing revealed that both *bla*_VIM-84_ (GenBank accession: ON688661.1) and *bla*_VIM-85_ (GenBank accession: ON688662.1) had a length of 801 bp and they were highly similar to bla_VIM-24_ (GenBank accession: HM855205), with nucleotide identities of 99.88 and 99.75%, respectively (Wang et al., 2023). In this study, according to BLASTn search, *bla*_VIM-92_ was highly similar to *bla*_VIM-84_ and *bla*_VIM-85_, with a nucleotide identity of 99.63 and 99.5%, respectively. Therefore, we selected the plasmids pWTJH2 (GenBank accession: CP104585.1), pWTJH6 (GenBank accession: CP104587.1), and pPJK40 to compare the genetic backgrounds of the VIM alleles in *P. aeruginosa* (Figure 4).

In plasmids pWTJH2 and pWTJH6, the *bla*_VIM-84_ and *bla*_VIM-85_ genes are embedded within class 1 integrons. Similarly, in the plasmid pPJK40, the *bla*_VIM-92_ gene is also embedded in a class 1 integron. The *bla*_VIM-84_ gene is the second cassette in a class 1 integron, with the fosE gene located upstream. The downstream cassettes include aac(6′)-Ib4, *bla*_OXA-101_, and ant1 within an integron that originally carried *bla*_VIM-24_. In this integron, the qacEΔ1 and sul1 genes in the 3′ conserved segment (3’-CS) were replaced by an IS26 element.

For the *bla*_VIM-85_ genetic environment, the 3’-CS is interrupted by a gene encoding GNAT family N-acetyltransferase, clipped by the insertion of transposon Tn*5393*. A similar genetic structure was observed in *bla*_VIM-92_, characterized by the arrangement intI-aac(6′)-Ib4-*bla*_VIM-92_-qacEΔ1-sul1. In this configuration, *bla*_VIM-92_ is embedded within the second gene cassette of a class 1 integron, with the aac(6′)-Ib4 resistance gene positioned upstream. In particular, the downstream *bla*_OXA-101_ and ant1 genes are absent. The integron harboring *bla*_VIM-92_ was inserted into transposon Tn*1403* (GenBank accession: AF313472.2), which contains a backbone with the genes tnpA, tnpR, and orfABCD, along with a class 1 integron and Tn*5393*. In Tn*5393*, the tnpA gene is interrupted by an IS66 family insertion (Stokes et al., 2007).

## 4 Discussion

*Pseudomonas aeruginosa* is capable of quickly evolving and developing resistance to adapt to environmental conditions, especially in hospital settings. For example, studies have shown that after prolonged exposure in different hospital environments, *P. aeruginosa* adapts to the host environment, modulating the expression of numerous virulence factors and acquiring or developing mechanisms for antibiotic resistance, including resistance to carbapenems (Cameron et al., 2022). This adaptability is a major factor contributing to the frequent occurrence of hospital-acquired infections. In China, surveillance data from 2021 reported that *P. aeruginosa* accounted for 7.96% of hospital-acquired infections, highlighting its significant role in clinical settings (Yan et al., 2022). Among these, CRPA has become increasingly prevalent due to the widespread use of antibiotics. CRPA poses a major threat to public health, with its rising detection rates associated with increased morbidity and mortality (Litwin et al., 2020). Furthermore, CRPA exhibits resistance not only to carbapenems but also to multiple classes of antibiotics, severely limiting treatment options (Ning and Yang, 2022). The extensive dissemination of CRPA and its ability to transfer resistance genes to other bacteria underscore the need for effective surveillance and control measures to curb its spread and impact.

In this study, the average annual CRPA detection rate in the First Affiliated Hospital of Hebei North University from 2021 to 2023 was 16.55%, derived by averaging the overall detection rate of 46.95% over 3 years, which was comparable to the national detection rate reported for 2022 (16.6%), but slightly lower than the rate for Hebei Province in the same year (17.4%) (Yu et al., 2022). With the widespread use of antibiotics, the detection rate of *P. aeruginosa* resistant to multiple antibiotics is increasing yearly (Litwin et al., 2020). Due to the high mortality rate, propensity for cross-infection, and difficulty in treatment, the emergence of CRPA had made infection control in hospitals increasingly difficult. Although the detection rate of CRPA may differ across regions, its higher detection rate in specific areas, especially within local hospital environments, emphasizes the critical need for focused surveillance and control strategies (Huang et al., 2023). This difference in detection rates may be related to factors such as the hospital classification level, patient demographics, regional conditions, and the impact of the COVID-19 pandemic. In the present work, 71 CRPA isolates mainly came from four key departments: respiratory, the others, international medical department and the ICU. Notably, the department distribution of CRPA is largely consistent with previous reports (Li et al., 2021). Patients in these departments are often critically ill, require prolonged hospital stays, frequently undergo invasive procedures (e.g., tracheal intubation and tracheostomy), leading to Device-associated Hospital-acquired Infections (DA-HAIs) in some patients, and have multiple underlying conditions. Related studies suggested that the incidence of infections in ICU patients might have varied across different geographical regions, hospitals, and even among different ICUs within the same hospital. The types of infections, the profiles of pathogens causing these infections, and their antimicrobial susceptibility patterns also varied depending on the location (Alfouzan et al., 2021). These factors collectively facilitated the spread of CRPA. Long-term use of antibiotics and immunosuppressants compromises immune function, increasing susceptibility to CRPA infection (Rossi et al., 2021; Pettigrew et al., 2019). Many studies showed that inappropriate and irrational use of antibiotics to treat infections led to the emergence of multidrug-resistant (MDR) isolates of common bacterial isolates (Rizzo et al., 2019). This results in prolonged hospital stays, significantly increased morbidity and mortality, and contributes to the spread of CRPA. These findings demonstrate the importance of judicious use of antibiotics following antimicrobial guidelines. When performing invasive procedures on CRPA patients, strict aseptic protocols, improved surface disinfection, timely equipment replacement, and early tube removal are crucial to prevent infection. Subsequently, statistical analysis was conducted on the types of specimens, and it was found that most CRPA isolates were detected in sputum specimens, accounting for 77.46%, which is consistent with reports that CRPA is mainly strained from sputum (Gill et al., 2022). However, as *P. aeruginosa* is part of normal respiratory flora, it can quickly colonize the respiratory tract as an opportunistic pathogen. Thus, interpreting the results of respiratory cultures requires careful assessment to confirm infection. Previous research (Yan et al., 2022) has linked CRPA strained from sputum to higher long-term mortality, indicating that it may be a potential source of hospital-acquired infections that warrant attention.

In recent years, the detection rate of MBLs has increased significantly, with a broad distribution across bacterial species and geographic regions, making them a focal point of clinical concern (Safavi et al., 2020). MBL genes can reside on integrons, transposons, plasmids, chromosomes, or other genetic elements, and their plasmid-mediated mobility improves transferability, contributing to bacterial resistance. Clinically, carbapenems such as imipenem, meropenem, ertapenem, and doripenem are ineffective against MBLs, except for monobactams (Bush, 2001). Therefore, the presence of MBLs is likely to increase the risk of treatment failure in carbapenem-resistant *P. aeruginosa* infections. The increasing prevalence of MBLs highlights their growing significance in hospital-acquired infections, further emphasizing the urgent need for effective surveillance and control strategies to mitigate their public health impact. Among acquired MBL genotypes, blaVIM is the most prevalent, with multiple variants differentiated by gene and translated amino acid sequences. In this study, VIM-producing isolates accounted for 1.40% (2/143) of all isolates, with VIM-92-producing isolates belonging to the sequence type ST207. Although the global prevalence of ST207 may be lower than classic sequence types such as ST235 and ST111, it has been reported in some regions as an MDR isolate carrying carbapenemase genes, such as blaVIM or blaNDM (Gondal et al., 2024). ST244 CRPA was consistently detected in the same hospital over three consecutive years. This observation suggested the dissemination of ST244 isolates producing OXA-101 and PER-1 enzymes within the hospital. In the analysis of T3SS virulence factors, we identified an isolate, PJK15, which carried four virulence factors. Previous studies had generally suggested that *exoU* and *exoS* were mutually exclusive due to occupying the same chromosomal locus (Freschi et al., 2019). However, recent studies have revealed that high-risk clones co-carrying the T3SS effector genes *exoS* and *exoU* are capable of causing community-acquired infections (CAIs) in non-immunocompromised young individuals, likely due to the enhanced virulence profiles of these isolates (Song et al., 2023). In our study, we hypothesized that the coexistence of these virulence factors in PJK15 was induced by mutations triggered by environmental pressures on the isolate. The simultaneous presence of both virulence factors could potentially influence the severity of the infection and the response to treatment, highlighting the urgent need for further investigation.

We identified two *P. aeruginosa* isolates, PJK40 and PJK43, carrying the *bla*_VIM-92_ gene and selected PJK40 for further study in this research. This isolate exhibited elevated MICs for β-lactams, cephalosporins, and β-lactam combination agents, attributed to acquiring the resistant plasmid pPJK40. The *bla*_VIM-92_ gene is located on a pJB37-like megaplasmid of the IncP-2 family. Typically, plasmids acquire resistance genes through mobile elements, allowing their spread within and between bacterial species (Hall et al., 2022). However, the results of our conjugation experiments indicated that pPJK40 was not conjugative, likely due to its specific structural configuration, which includes the *bla*_VIM-92_ resistance gene. Antimicrobial susceptibility testing comparing PAO1 MICs expressing *bla*_VIM-92_ and *bla*_VIM-24_ indicated that *bla*_VIM-92_ confers similar or, in some cases, higher resistance to most antibiotics, except for aztreonam. This suggests that VIM-92 may be equally or more effective than VIM-24 in mediating antibiotic resistance.

In conclusion, with its unique geographic location, our study in Zhangjiakou identified 71 CRPA isolates and analyzed CRPA resistance trends in certain regions of northern China from 2021 to 2023. Among the CRPA isolates, two produced the novel metallo-β-lactamase, VIM-92. *bla*_VIM-92_ was in a pJB37-like plasmid within a distinct genetic context compared to other VIM alleles in *P. aeruginosa*. We also identified an isolate carrying four T3SS virulence factors simultaneously. With the continuous emergence of carbapenemase-producing *P. aeruginosa*, these findings highlight the importance of ongoing surveillance and comparative studies to inform effective antimicrobial stewardship and public health interventions in the northern region.

## Data Availability

The datasets presented in this study can be found in online repositories. The names of the repository/repositories and accession number(s) can be found at: https://www.ncbi.nlm.nih.gov/genbank/, PQ563311; https://www.ncbi.nlm.nih.gov/genbank/, ON688661.1; https://www.ncbi.nlm.nih.gov/genbank/, HM855205; https://www.ncbi.nlm.nih.gov/genbank/, CP104585.1; https://www.ncbi.nlm.nih.gov/genbank/, CP104587.1; https://www.ncbi.nlm.nih.gov/genbank/, AF313472.2; https://www.ncbi.nlm.nih.gov/genbank/, KY494864.1; https://www.ncbi.nlm.nih.gov/genbank/, CP104591.1; https://www.ncbi.nlm.nih.gov/genbank/, MT732194.1; https://www.ncbi.nlm.nih.gov/genbank/, ON688662.1.

## Footnotes

^1^ https://github.com/tseemann/shovill

^2^ https://pubmlst.org/organisms/pseudomonas-aeruginosa

^3^ https://github.com/tseemann/abricate

^4^ http://www.softberry.com
