# Supplementary material for: High prevalence of carbapenem-resistant Pseudomonas aeruginosa and identification of a novel VIM-type metallo-β-lactamase, VIM-92, in clinical isolates from northern China
Source: Front Microbiol. 2025 Feb 26;16:1543509. doi: 10.3389/fmicb.2025.1543509 (PMC11897005; doi:10.3389/fmicb.2025.1543509)
Supplement: Supplementary file 1 [file Data_Sheet_1.docx]

Supplementary Material

# Supplementary Figures and Tables

## Supplementary Figures


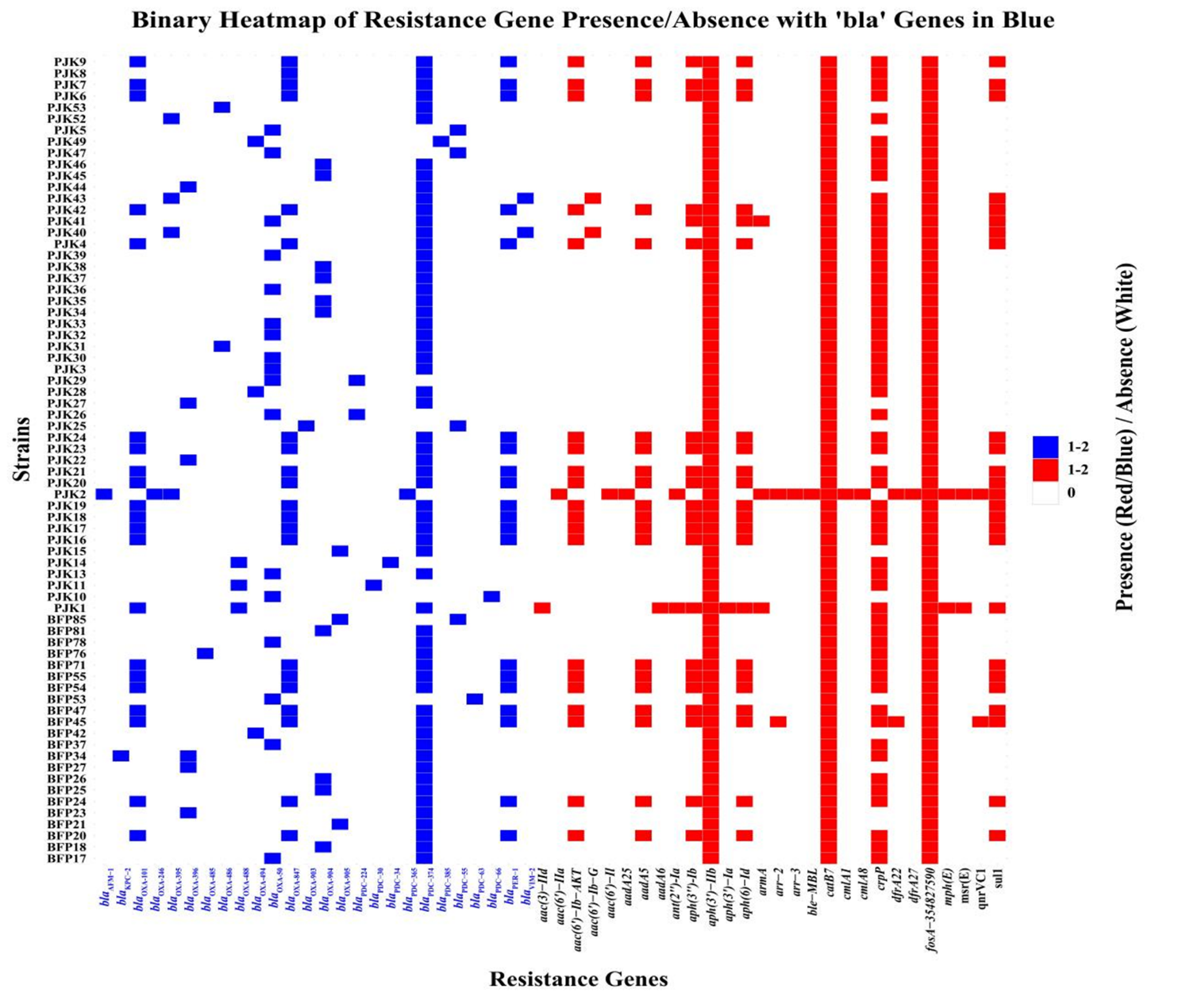


**Supplementary Figure S1.** The heatmap illustrates the presence (colored) and absence (white) of resistance genes in 71 CRPA strains. Resistance genes are displayed along the x-axis, while bacterial strains are listed along the y-axis. The binary data represent the presence (1 or 2) or absence (0) of the genes.


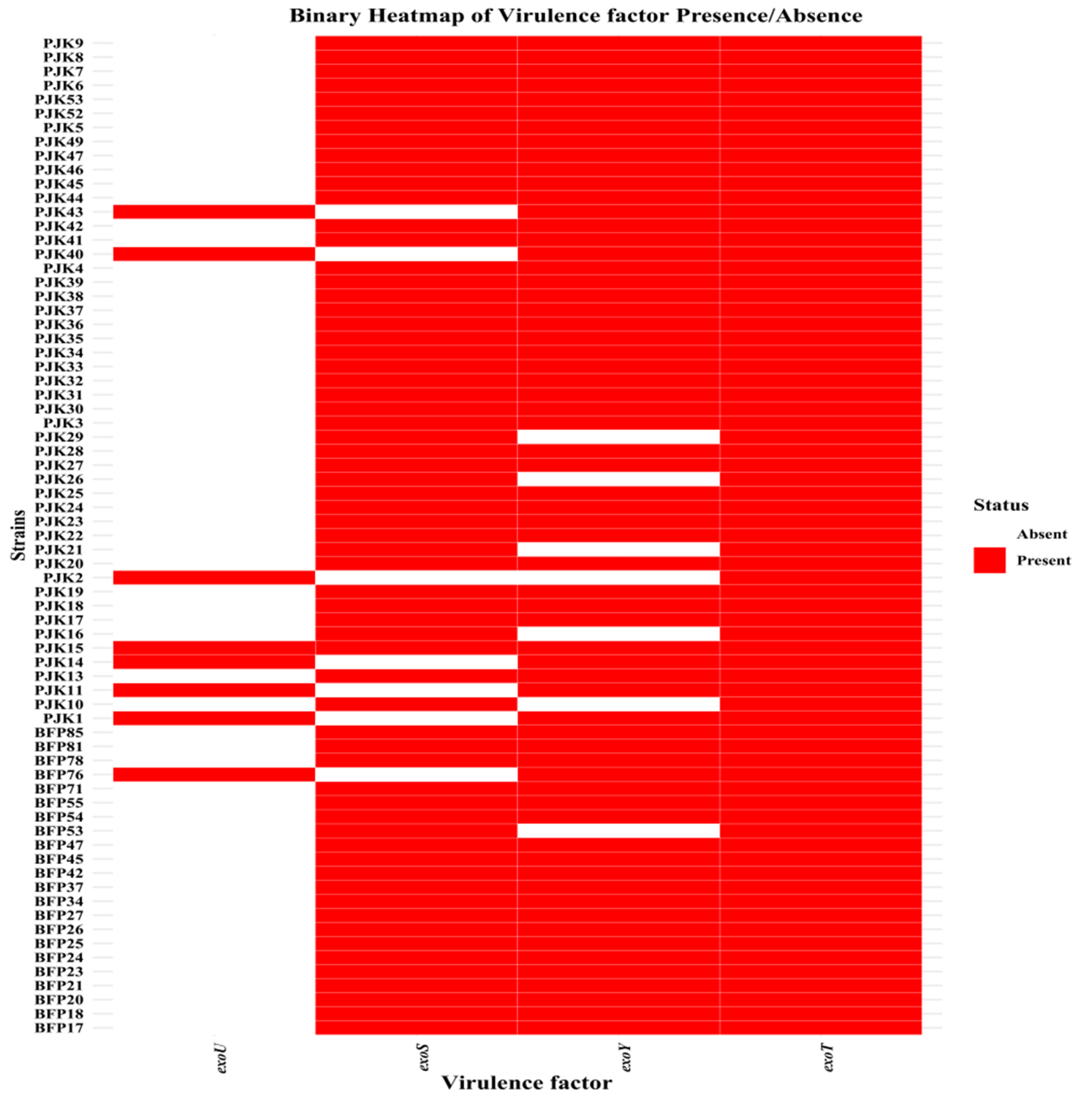


**Supplementary Figure S2.** The heatmap illustrates the presence (red) and absence (white) of virulence factor in 71 CRPA strains. Virulence factor are displayed along the x-axis, while bacterial strains are listed along the y-axis.


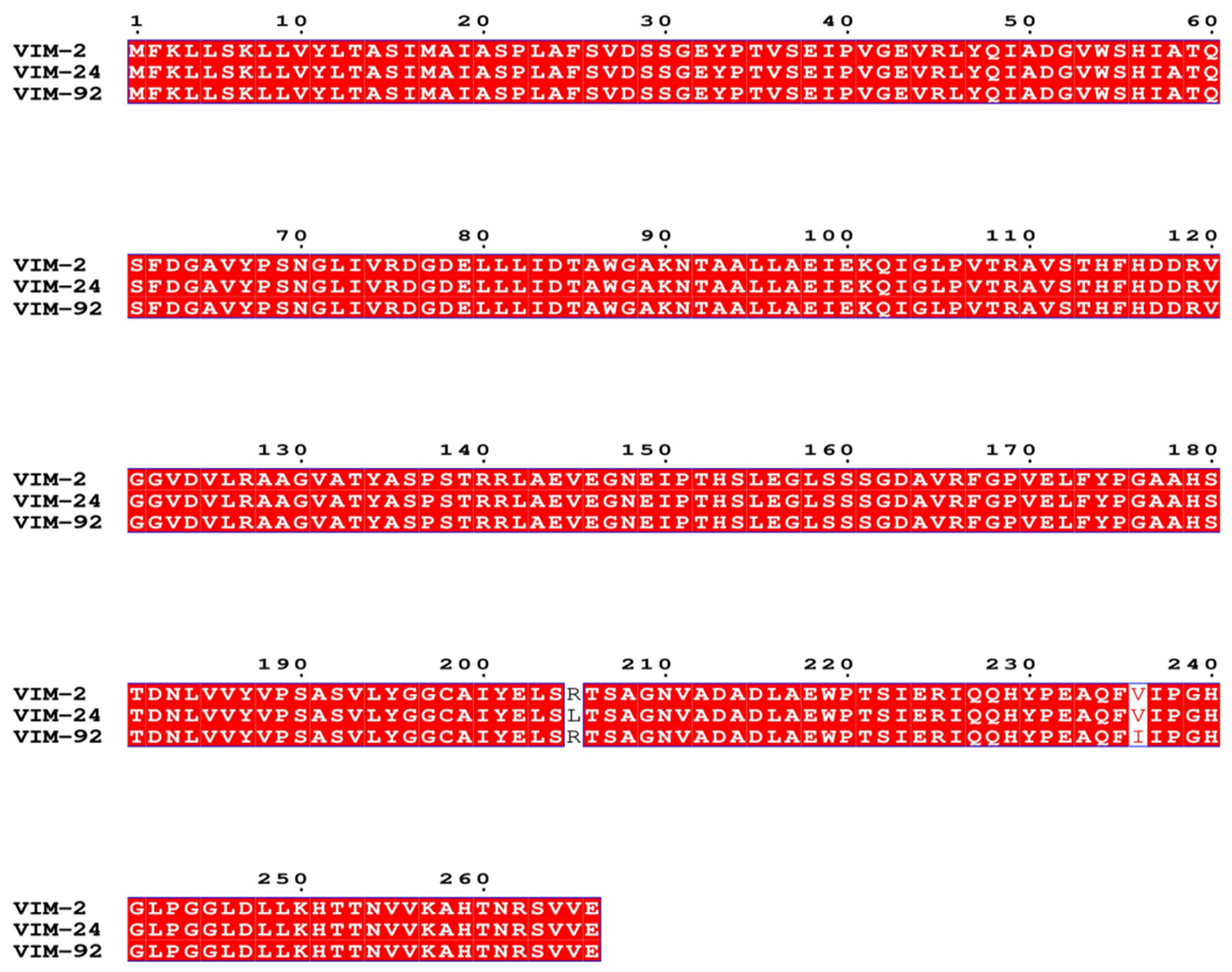


**Supplementary Figure S3.** The Amino acid sequence alignment of VIM-2, VIM-24, and VIM-92

## Supplementary Tables

**Supplementary Table S1.** The isolation rates of CRPA strains from 2021 to 2023

| **Year** |  | **PA (isolates)** |  | **CRPA (isolates)** |  | **CRPA Detection Rate（%）** |
| --- | --- | --- | --- | --- | --- | --- |
| 2021 |  | 25 |  | 24 |  | 96% |
| 2022 |  | 32 |  | 25 |  | 78.13% |
| 2023 |  | 86 |  | 22 |  | 25.58% |
| Total |  | 143 |  | 71 |  | 49.65% |

**Supplementary Table S2.** The isolation rates of CRPA strains from 2021 to 2023

| **Department** | **2021 (n=24)** | |  | **2022 (n=25)** | |  | **2023 (n=22)** | |  | **Total (n=71)** | |
| --- | --- | --- | --- | --- | --- | --- | --- | --- | --- | --- | --- |
|  | **Strain count** | **Proportion (%)** |  | **Strain count** | **Proportion (%)** |  | **Strain count** | **Proportion (%)** |  | **Strain count** | **Proportion (%)** |
| Respiratory | 7 | 29.17 |  | 6 | 24 |  | 5 | 22.73 |  | 18 | 25.35 |
| Icu | 3 | 12.5 |  | 9 | 36 |  | 1 | 4.55 |  | 13 | 18.31 |
| Neurosurgery | 3 | 12.5 |  | 2 | 8 |  | 1 | 4.55 |  | 6 | 8.45 |
| International Medical Department | 6 | 25 |  | 1 | 4 |  | 7 | 31.82 |  | 14 | 19.72 |
| Others | 5 | 20.83 |  | 7 | 28 |  | 8 | 36.36 |  | 20 | 28.17 |

**Supplementary Table S3.** Distribution of specimen sources for CRPA strain isolation (2021-2023)

| **Sample Type** | **2021 (n=24)** | |  | **2022 (n=25)** | |  | **2023 (n=22)** | |  | **Total (n=71)** | |
| --- | --- | --- | --- | --- | --- | --- | --- | --- | --- | --- | --- |
|  | **Strain count** | **Proportion (%)** |  | **Strain count** | **Proportion (%)** |  | **Strain count** | **Proportion (%)** |  | **Strain count** | **Proportion (%)** |
| Sputum | 18 | 75 |  | 20 | 80 |  | 17 | 77.27 |  | 55 | 77.46 |
| Secretions | 2 | 8.33 |  | 1 | 4 |  | 1 | 4.55 |  | 4 | 5.63 |
| Urine | 1 | 4.17 |  | 0 | 0 |  | 1 | 4.55 |  | 2 | 2.82 |
| Lavage fluid | 2 | 8.33 |  | 3 | 12 |  | 0 | 0 |  | 5 | 7.04 |
| Others | 1 | 4.17 |  | 1 | 4 |  | 3 | 13.64 |  | 5 | 7.04 |
